# Reproducibility of exhaled nitric oxide measurements in overweight and obese adults

**DOI:** 10.1186/1756-0500-7-775

**Published:** 2014-11-03

**Authors:** Willemien Thijs, Renée de Mutsert, Saskia le Cessie, Pieter S Hiemstra, Frits R Rosendaal, Saskia Middeldorp, Klaus F Rabe

**Affiliations:** Department of Pulmonology, Leiden University Medical Center, PO Box 9600, Leiden, 2300 RC the Netherlands; Department of Clinical Epidemiology, Leiden University Medical Center, Leiden, the Netherlands; Department of Medical Statistics, Leiden University Medical Center, Leiden, the Netherlands; Department of Vascular Medicine, Academic Medical Center, Amsterdam, the Netherlands; LungenClinic Grosshansdorf, Grosshansdorf, Germany

**Keywords:** Reproducibility, Exhaled nitric oxide, Obesity

## Abstract

**Background:**

Exhaled nitric oxide is a noninvasive measure of airway inflammation that can be detected by a handheld device. Obesity may influence the reproducibility of exhaled nitric oxide measurements, by - for instance – decreased expiratory reserve volume.

**Findings:**

We analyzed triple exhaled nitric oxide measurements from 553 participants (aged 45 to 65 years with a body mass index ≥27 kg/m^2^) of the Netherlands Epidemiology of Obesity Study. The interclass correlation coefficient (single measurement reliability) was 0.965 (95% CI: 0.960, 0.970).

**Conclusions:**

We conclude that for assessment of exhaled nitric oxide in large cohorts of overweight and obese adults a single measurement suffices.

## Findings

### Introduction

Exhaled nitric oxide (eNO) is a noninvasive marker of inflammation in the airways. The levels of eNO correlate well with other markers of inflammation in the airways of asthmatics, such as sputum eosinophils and airway eosinophilia in bronchial biopsies [1, 2]. Measuring eNO with a handheld device is a convenient way to assess airways inflammation and has been used to study e.g. occupational hazards or asthma [3, 4]. The prevalence of obesity has risen dramatically in the past decades and an increasing proportion of participants in studies will be overweight or obese [5]. Because eNO measurements take time and generate costs it is important to establish the reproducibility of eNO measurements in overweight and obese adults.

How could obesity influence eNO measurements? Obesity is associated with a loss in expiratory reserve volume [6], which may influence eNO measurement that require a slow and steady exhalation. In addition, obesity is associated with low grade systemic inflammation [7] which may be accompanied by airways inflammation resulting in increased eNO levels. However, studies into the association between obesity and levels of eNO show conflicting results [8–11]. Therefore it is not clear whether putatively increased eNO levels may contribute to decreased reproducibility in obese subjects.

The ATS/ERS recommendations for eNO measurements suggest two measurements of eNO [12]. Because of the time requirement and costs associated with multiple eNO measurements in large scale studies, a single measurement would be preferable. Reproducibility of eNO measured by the handheld NIOX MINO has been evaluated in children [13], adults [14], asthma patients and pregnant women [15], but not in overweight and obese individuals. Therefore, we used a triplicate measurement to assess the reproducibility of eNO measured by a handheld NIOX MINO in a cohort study of overweight and obese adults, with the aim to assess whether a single measurement may suffice in large scale studies.

### Materials and methods

The Netherlands Epidemiology of Obesity (NEO) Study is a population-based cohort study in adults aged 45 to 65 years, with an oversampling of participants with overweight or obesity [16]. The study was approved by the ethical committee of the Leiden University Medical Center and all participants gave written informed consent. The present analysis includes the first 630 participants with a body mass index (BMI) ≥ 27 kg/m^2^. Completed multiple questionnaires including self-reported asthma, and anthropometric and maximal flow-volume curves measurements were obtained. Exhaled nitric oxide was measured using a portable analyzer, the NIOX MINO (Aerocrine AB, Solna, Sweden). Participants performed a 10 seconds slow steady exhalation. Three successive recordings at 1-minute intervals, expressed as parts per billion (ppb), were made. The interclass correlation coefficient (ICC) was calculated for the three measurements in all participants, participants with self reported asthma and separately for participants with a BMI ≥ 35 and for elevated mean eNO levels (>25 ppb and for >50) [17]. The mean intra-participant difference in eNO was calculated and a Bland-Altman plot was constructed. Statistical analyses were performed with SPSS 20.0 software (SPSS Inc., Chicago, IL).

### Results

**Figure 1:**
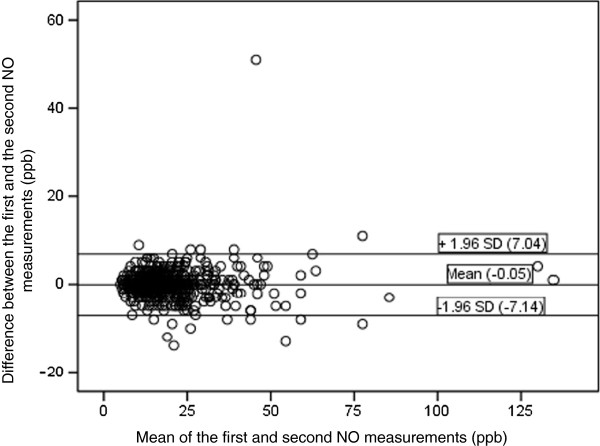
**Bland-Altman plot for the first two eNO measurements by the NIOX MINO (n = 553).** The dots represent the difference between the first and the second measurement.

Of the first 630 participants of the NEO study, 46 participants did not perform eNO measurements because they did not visit the lung function department due to logistic problems. In another 31 patients, no measurements were obtained because of inability to perform the technique or because of a technical failure with the nitric oxide machines. As a result, the present analysis includes 553 participants who performed all three eNO measurements. The characteristics of the study population and results of eNO measurements are presented in Table 1. The ICC (single measurement reliability) for all participants was 0.965 (95% CI: 0.960, 0.970), whereas it was 0.926 (95% CI: 0.926, 0.965) for the participants with a BMI ≥ 35 (n = 92). The ICC (single measurement reliability) for all participants with asthma (n = 39) was 0.988 (95% CI: 0.979, 0.993), whereas it was 0.932 (95% CI: 0.818, 0.981) for the participants with asthma and a BMI ≥ 35 (n = 10). The ICC for all eNO measurements that exceeded 25 ppb (n = 109) was 0.949 (95% CI: 0.931, 0.963) and for those that exceeded 50 ppb (n = 18) was 0.911 (95% CI: 0.818, 0.963). The mean intra-participant difference in eNO for all participants was for the second and first reading: -0.05 ppb (95% CI: -7.14, 7.04); third and first reading -0.15 ppb (95% CI: -6.8, 7.6); and third and second reading -0.13 ppb (95% CI: -5.9, 6.5). A Bland-Altman plot was constructed for the first two measurements (Figure 1).

**Table 1:** **Clinical characteristics and eNO measurements of the study population (n = 553)**

Characteristic	Median or %	IQR
Age (years)	56	(50-61)
Sex (women %)	47	NA
Self reported asthma (%)	7	NA
BMI (kg/m^2^)	30	(28-33)
FEV_1_ % predicted	103	(92-114)
FVC % predicted	105	(96-115)
First nitric oxide (ppb)	17	(12-23)
Second nitric oxide (ppb)	17	(12-24)
Third nitric oxide (ppb)	17	(13-24)

BMI: Body mass index; IQR: Interquartile range; NA: not applicable; FEV_1_ %: percent predicted of forced expiratory volume; FVC % percent predicted of forced vital capacity; ppb: parts per billion.

### Discussion

The ICC and mean intra-participant difference in eNO for all 553 participants was in line with previous reproducibility studies performed on the NIOX MINO in other populations [13, 14]. The ICC for participants with a BMI ≥ 35 kg/m^2^ was slightly lower (but clinically not relevant) than within the whole group, possibly as a result of decreased expiratory reserve volumes. Low grade inflammation associated with obesity appears a less likely explanation for the small loss in reproducibility because only early studies report a positive correlation between BMI and eNO [8, 9]; later studies have not been able to reproduce these initial findings [10, 11]. The reproducibility in participants with self reported asthma was in line with the results in the whole group but within our study the reproducibility at higher eNO levels was slightly lower. In earlier study by Selby et al. [13] it is concluded that for individual absolute levels two measurements are needed. Similarly, we conclude that in clinical practice two eNO measurements are advised but despite small differences of ICC in different analyses, our results demonstrate that in large cohorts of overweight and obese adults a single eNO measurement suffices, which will have significant logistical and financial consequences for cohort studies.

## Abbreviations

BMI: Body mass index
ICC: Inter class correlation
eNO: Exhaled nitric oxide
Ppb: Parts per billion
NEO: Netherlands Epidemiology of Obesity
FEV_1_%: Percent predicted of forced expiratory volume
FVC%: Percent predicted of forced vital capacity
ATS: American Thoracic Society
ERS: European Respiratory Society.

## References

[1] Jatakanon A, Lim S, Kharitonov SA, Chung KF, Barnes PJ (1998). Correlation between exhaled nitric oxide, sputum eosinophils, and methacholine responsiveness in patients with mild asthma. Thorax.

[2] Payne DN, Adcock IM, Wilson NM, Oates T, Scallan M, Bush A (2001). Relationship between exhaled nitric oxide and mucosal eosinophilic inflammation in children with difficult asthma, after treatment with oral prednisolone. Am J Respir Crit Care Med.

[3] Sordillo JE, Webb T, Kwan D, Kamel J, Hoffman E, Milton DK, Gold DR (2011). Allergen exposure modifies the relation of sensitization to fraction of exhaled nitric oxide levels in children at risk for allergy and asthma. J Allergy Clin Immunol.

[4] Tungu AM, Bratveit M, Mamuya SD, Moen BE (2013). Fractional exhaled nitric oxide among cement factory workers: a cross sectional study. Occup Environ Med.

[5] WHO (2000). Obesity, Preventing and Managing the Global Epidemic. Report of a WHO Consultation.

[6] Littleton SW (2012). Impact of obesity on respiratory function. Respirology.

[7] Tilg H, Moschen AR (2008). Role of adiponectin and PBEF/visfatin as regulators of inflammation: involvement in obesity-associated diseases. Clin Sci (Lond).

[8] Depalo A, Carpagnano GE, Spanevello A, Sabato R, Cagnazzo MG, Gramiccioni C, Foschino-Barbaro MP (2008). Exhaled NO and iNOS expression in sputum cells of healthy, obese and OSA subjects. J Intern Med.

[9] Maestrelli P, Ferrazzoni S, Visentin A, Marian E, Dal BD, Accordino R, Fabbri LM (2007). Measurement of exhaled nitric oxide in healthy adults. Sarcoidosis Vasc Diffuse Lung Dis.

[10] Kim SH, Kim TH, Lee JS, Koo TY, Lee CB, Yoon HJ, Shin DH, Park SS, Sohn JW (2011). Adiposity, adipokines, and exhaled nitric oxide in healthy adults without asthma. J Asthma.

[11] Lombardi C, Gargioni S, Gardinazzi A, Canonica GW, Passalacqua G (2011). Impact of bariatric surgery on pulmonary function and nitric oxide in asthmatic and non-asthmatic obese patients. J Asthma.

[12] **ATS/ERS recommendations for standardized procedures for the online and offline measurement of exhaled lower respiratory nitric oxide and nasal nitric oxide, 2005** *Am J Respir Crit Care Med* 2005, **171:**912–930. 10.1164/rccm.200406-710ST 15817806

[13] Selby A, Clayton B, Grundy J, Pike K, Drew K, Raza A, Kurukulaaratchy R, Arshad SH, Roberts G (2010). Are exhaled nitric oxide measurements using the portable NIOX MINO repeatable?. Respir Res.

[14] Alving K, Janson C, Nordvall L (2006). Performance of a new hand-held device for exhaled nitric oxide measurement in adults and children. Respir Res.

[15] Tamasi L, Bohacs A, Bikov A, Andorka C, Rigo J, Losonczy G, Horváth I (2009). Exhaled nitric oxide in pregnant healthy and asthmatic women. J Asthma.

[16] De MR, Den HM, Rabelink TJ, Smit JW, Romijn JA, Jukema JW, de Roos A, Cobbaert CM, Kloppenburg M, le Cessie S, Middeldorp S, Rosendaal FR (2013). The Netherlands Epidemiology of Obesity (NEO) study: study design and data collection. Eur J Epidemiol.

[17] Taylor DR, Pijnenburg MW, Smith AD, De Jongste JC (2006). Exhaled nitric oxide measurements: clinical application and interpretation. Thorax.

